# Shaping the future of radiation oncology education: results of a nationwide student survey from Germany

**DOI:** 10.1007/s00066-025-02493-x

**Published:** 2026-01-07

**Authors:** Hendrik Dapper, Michael Oertel, Hilke Vorwerk, Sophia Drabke, Diana Steinmann, Emmanouil Fokas, Heinz Schmidberger, Nanna E. Wielenberg, Matthias Mäurer, Philipp Linde

**Affiliations:** 1 Department of Radiation Oncology, Cyberknife and Radiation Therapy, Center for Integrated Oncology Aachen Bonn Cologne Duesseldorf (CIO ABCD), Faculty of Medicine and University Hospital of Cologne, University of Cologne, Cologne, Germany; 2 Department of Radiation Oncology, University Hospital Münster, Münster, Germany; 3 West German Cancer Center Münster (WTZ), University Hospital Münster, Münster, Germany; 4 Department of Radiation Therapy and Oncology, UKGM GmbH, University Hospital Marburg, Faculty of Medicine, Philipps-University of Marburg, Marburg, Germany; 5 Department of Radiation Oncology, University Medical Center Mainz, Mainz, Germany; 6 Department of Radiotherapy, Hannover Medical School, Hannover, Germany; 7 Department of Radiation Oncology, Medical Center University of Freiburg, Medical Faculty University of Freiburg, Freiburg, Germany; 8 Department of Radiation Oncology, Jena University Hospital, Jena, Germany

**Keywords:** Radiation therapy, Medical education, Digital learning, Students’ perspective, Curriculum development

## Abstract

**Background and purpose:**

Radiation oncology (RO) is a key component of cancer care and should be adequately represented in undergraduate medical education. In light of medical training reforms in German medical education and rising expectations for competency-based, practice-oriented learning, this nationwide survey explored medical students’ perspectives on RO teaching and its future development.

**Materials and methods:**

A cross-sectional online survey was conducted from 2023 to 2025 across all 38 German medical faculties. Developed by the Working Group on Teaching of the German Society for Radiation Oncology (DEGRO), the 49-item questionnaire addressed curriculum structure, teaching quality, preferred formats, and interest in RO.

**Results:**

Among 1112 participants, 739 completed the survey. Although 96% viewed RO as being essential to cancer care, 62% felt it was underrepresented in the curriculum. Students called for more interdisciplinary and patient-centered teaching and showed high interest in digital and hybrid learning formats. Notably, 60.5% favored interdisciplinary oncology formats including RO, and nearly half expressed interest in elective RO courses.

**Conclusion:**

Despite its recognized importance, RO remains inconsistently represented in medical education. Embedding RO in interdisciplinary oncology modules and expanding digital, longitudinal, and flexible teaching formats could enhance its visibility and relevance. Given students’ strong interest in more in-depth exposure, optional and elective formats should be further developed to meet modern educational expectations.

**Supplementary Information:**

The online version of this article (10.1007/s00066-025-02493-x) contains supplementary material, which is available to authorized users.

## Introduction

In Germany, over 450,000 people are diagnosed with cancer each year, and around half will require radiotherapy (RT) [1]. Radiation therapy is one of the three main pillars of modern cancer care, alongside surgery and systemic therapies, making radiation oncology (RO) knowledge a critical competency for all physicians [2]. This knowledge is essential not only for future oncologists but also for other physicians to appropriately counsel patients and contribute to multidisciplinary cancer care [3].

Although RO is often seen as underrepresented in curricula, students’ perspectives remain largely unknown [4]. RO is usually embedded in broader modules, and fewer than 10% of medical study programs include a dedicated RO examination [4, 5]. Practical RO exposure is limited: clerkships are often optional, and many schools offer no hands-on training [6]. As a result, graduates may leave with superficial knowledge and misconceptions about the scope and efficacy of RO [7]. In Europe, RO teaching has been described as inconsistent and undervalued, lagging behind other oncology specialties in quantity and visibility [8]. While the European Society for Radiotherapy and Oncology (ESTRO) School has provided postgraduate education for decades, undergraduate exposure remains largely unaddressed in the literature, underscoring the need for research into medical student education in this field [9, 10].

In Germany, these shortcomings are especially apparent: RO teaching is often limited to a few late-semester lectures or seminars, practical formats (e.g., bedside teaching or internships) are not mandatory, and fewer than half of faculties incorporate oncology content longitudinally [11]. These gaps raise concerns that graduates may undervalue the role of RT in patient care. Surveys confirm that RO is considered an integral component of oncology education [12, 13]. The number of dedicated teaching hours is often limited according to curriculum reviews, which may influence career interest—although student perceptions of adequacy remain not fully explored [14].

Recent reforms in German medical education present a timely opportunity to address these deficiencies. Germany’s *Masterplan Medizinstudium* and the revised Licensing Regulation for Physicians (*Ärztliche Approbationsordnung*, ÄApprO) mandate a shift from theory-based to competency-based learning, with greater emphasis on practical skills, interdisciplinary modules, and longitudinal learning experiences [15–17]. The National Competence-Based Learning Objectives Catalogue for Medicine 2.0 (*Nationaler Kompetenzbasierter Lernzielkatalog 2.0.*, NKLM), reflecting this shift, explicitly includes RT and interdisciplinary oncologic care, with specific competency goals for graduates [11]. About 70–80% of the new national core curriculum has compulsory content, expected to standardize oncology teaching nationwide. These reforms lay the groundwork for stronger integration of RO in undergraduate education [11, 18]. However, their implementation and impact on teaching remain to be evaluated.

Professional organizations have issued guidelines to strengthen RO education. The German Society for Radiation Oncology (DEGRO) convened a consortium that outlined recommendations for integrating RO into undergraduate curricula [11]. This consensus calls on all faculties to establish dedicated RO teaching, covering core topics (e.g., radiobiology, radiation physics) and ensuring a minimum exposure for every student [11]. Meanwhile, a focus group found that students support a longitudinal, practice-oriented oncology curriculum with earlier introduction of RT, and a single-center study showed that a preclinical RO course significantly improved student knowledge and interest [12, 13]. These insights align with the national trend toward more interdisciplinary, patient-centered teaching and underscore the need to modernize RO education.

No nationwide study has yet captured medical students’ perspectives on RO education. Most evaluations have been faculty led or performed in a single institution, leaving a gap in understanding students’ experiences across universities. To address this, a nationwide survey was conducted to assess RO teaching, evaluate its adequacy, and identify areas for improvement. By centering student perspectives, the study aims to inform curriculum development and support further integration of RO into undergraduate medical training.

## Methods

This nationwide, web-based cross-sectional survey assessed the structure, content, and perceived quality of undergraduate RO education across all 38 German medical faculties. It was conducted over three academic terms (winter 2023/24 to winter 2024/25) and included students who had completed most of their RO curriculum.

The questionnaire was developed by the DEGRO Working Group on Teaching, consisting of 12 RO educators and early-career professionals. Based on NKLM 2.0, the draft ÄApprO, and DEGRO priorities, items were created through a multistage consensus process and pilot-tested for clarity, validity, and feasibility.

The final survey comprised 49 items across seven thematic domains (e.g., RO content, teaching formats, clinical topics, basic sciences, and student interest), using mixed question types and adaptive logic. It was hosted on the GDPR-compliant platform UmfrageOnline (Online®, Enuvo Inc., Switzerland). Recruitment was coordinated via student councils, university networks, and national conferences. Participation was anonymous and voluntary, with digital informed consent obtained beforehand.

Quantitative data were analyzed descriptively; qualitative responses underwent thematic analysis by at least three reviewers. The study followed the Declaration of Helsinki and was approved by the ethics committee of the University of Jena (no. 123456789). Detailed methodological information is available in the Supplementary Material.

## Results

A total of 1112 individuals participated in the online survey, with 739 questionnaires (66.5%) completed in full. The average completion time was 10 min and 10 s. Most respondents were affiliated with university hospitals in Jena (29.3%), Mainz (24.4%), and Cologne (12.4%). The participant cohort primarily consisted of medical students who had already received teaching in RO and were predominantly in the advanced stages of their clinical training (Table 1).

**Table 1:** Participant characteristics

Participant characteristics, *N* = 1112	*n (%)*
*Sex*	
Female	716 (65.5)
Male	369 (33.8)
Diverse	4 (0.4)
No answer	4 (0.4)
*Type of study program*	
Standard study program	882 (81.0)
Model study course	200 (18.4)
Other	7 (0.6)
*Study stage*	
Preclinical	1 (0.001)
5th or 6th semester	25 (2.2)
7th or 8th semester	384 (34.5)
9th or 10th semester	611 (54.9)
Practical year	64 (5.8)
Licensed for a max. of 1 year	14 (1.3)
No statement	13 (1.2)
*Teaching during the COVID-19 pandemic*	
Yes	312 (28.7)
No	774 (71.3)

**Fig. 1:**
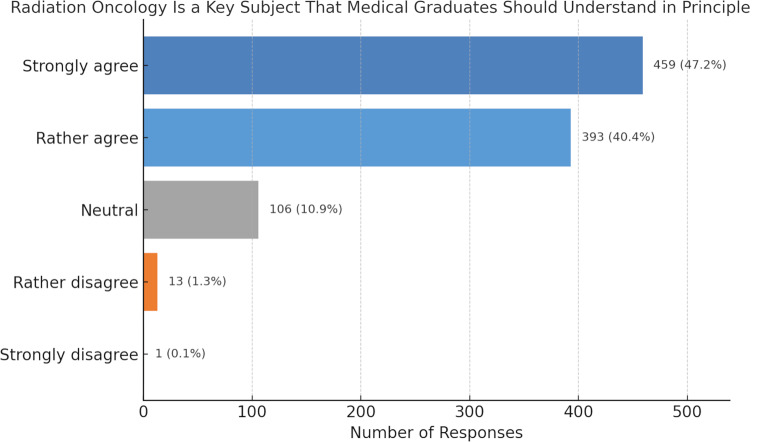
Bar chart with responses to the statement, “Radiation oncology is a key subject that medical graduates should understand in principle.”

**Fig. 2:**
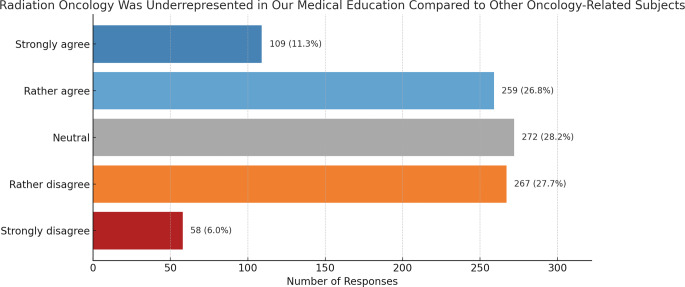
Bar chart with responses to the statement, “Radiation oncology was underrepresented in our medical education compared to other oncology-related subjects”

The vast majority of respondents (95.8%) either strongly or somewhat agreed that RO is essential for a comprehensive understanding of cancer patient care (Fig. 1). However, the perceived curricular presence of the subject was viewed more critically: a substantial proportion of respondents (61.9%) felt that radiation oncology is either partially or extensively underrepresented compared to other oncological disciplines (Fig. 2).

**Fig. 3:**
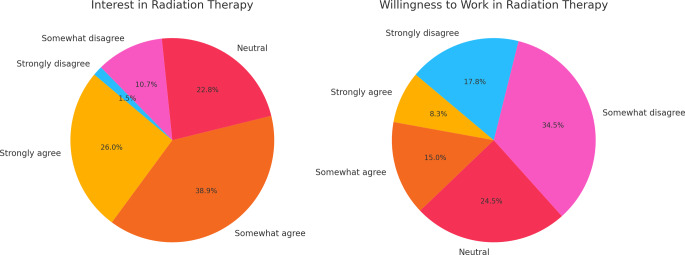
Pie chart showing responses regarding general interest in the field of radiation oncology and willingness to work in this profession

Overall interest in radiotherapy was high, with around 65% of respondents expressing some level of interest. Remarkably, 23% could envision pursuing a career in RO after graduation (Fig. 3).

**Fig. 4:**
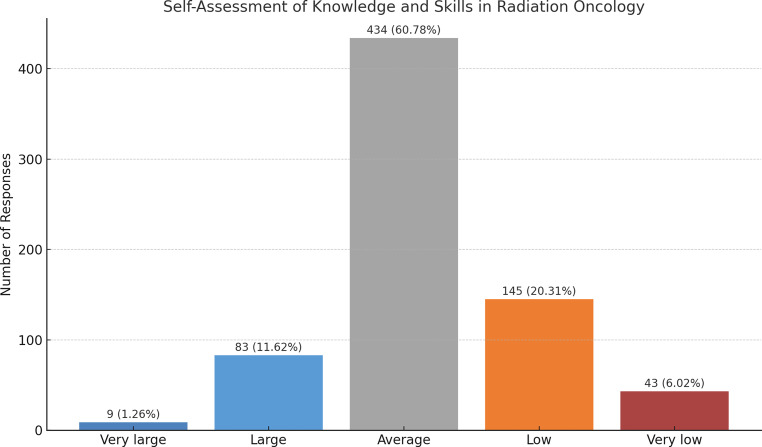
Bar chart showing the responses to the self-assessment of knowledge and skills in radiation oncology

Although most respondents were already at the end of their education, the majority rated their skills and knowledge in radiotherapy as average (60.8%). A proportion of 26.3% even rated their own skills as only low or very low (Fig. 4).

**Fig. 5:**
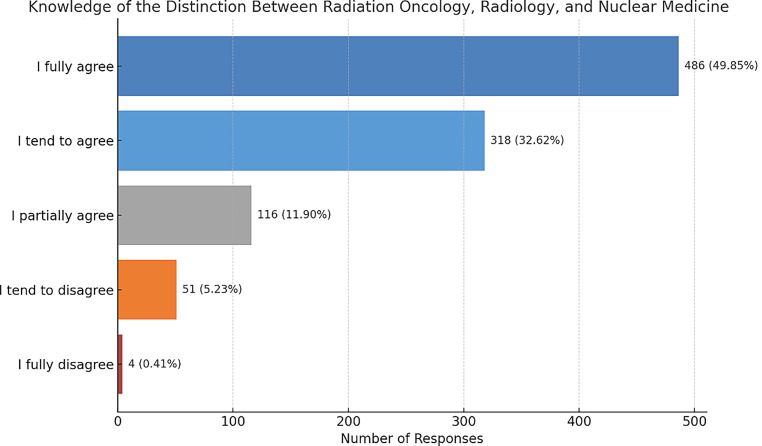
Bar chart showing the responses to the self-assessment of the ability to distinguish between radiation oncology, radiology, and nuclear medicine

Most students stated that they knew the differences between RO and the fields of radiology and nuclear medicine well or very well (82.5%; Fig. 5). A total of 77.1% of respondents reported that radiotherapy is primarily used for the treatment of malignant diseases, while 20.8% believed it is used equally for benign and malignant conditions. Only 0.4% assumed that it is mainly applied to benign diseases, and 1.8% indicated that they did not know.

**Fig. 6:**
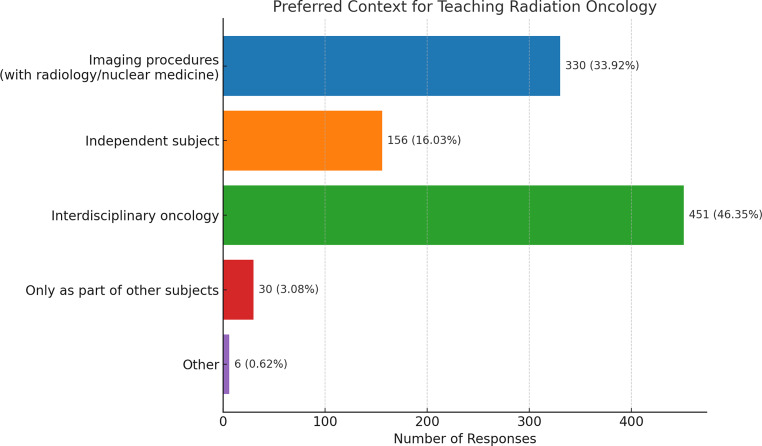
Bar chart showing responses to the preferred method of teaching radio-oncology content

Regarding the preferred context for teaching RO, the majority of respondents (46.4%) favored its integration into an interdisciplinary oncology curriculum. Approximately one third (33.9%) preferred a combined approach with radiology and nuclear medicine, while 16% supported it being taught as a standalone subject (Fig. 6). Only a small proportion believed it should be included merely as a supplementary topic (3.1%) or suggested other forms of integration (0.6%). The majority of respondents (76.9%) indicated that radiotherapeutic indications and treatments should be taught primarily in seminars or practical courses, while 19.9% preferred lectures. Only a small minority selected other formats (2.8%) or stated that the topic should not be taught at all (0.5%).

The medical students were asked about their preferred scope of RO teaching. On average, they wanted a total of around 19.3 teaching units (UE) of 45 min each for radiotherapy in medical studies. They would like the largest number of teaching units to be devoted to radiotherapy indications and specific therapies (Ø 7.32 UE), followed by patient-related teaching and discussion techniques (Ø 4.16 UE). They also attach relevant importance to scientific principles (Ø 4.01 UE) and clinical and technical principles (Ø 3.83 UE). This information reflects the desire for a practical and comprehensive understanding of radiotherapy. In terms of elective learning opportunities, the majority showed interest in interdisciplinary oncology formats involving radiotherapy (60.5%), followed by elective courses or block placements (45.3%) and clinical internships or observerships (27.6%). Approximately 18% expressed a general interest in further radiotherapy-related teaching formats.

The free-text responses from 666 medical students show that interest in radiation therapy can be increased primarily through practical, patient-oriented, and interactive teaching. More practical experience, insights into everyday clinical practice, structured and comprehensible teaching of the fundamentals, and interdisciplinary teaching formats were mentioned particularly frequently. Technical and physical content should be taught in a more application-oriented manner and better embedded in the overall medical context. In addition, many students would like transparent insights into professional practice, the reality of working life, and career paths in radiation therapy. This was illustrated by one student who noted, “I would have appreciated more clinical exposure to understand radiotherapy beyond physics lectures.”

## Discussion

The hereby presented analysis constitutes, to our knowledge, the largest survey on the state of medical education in RO, encompassing over 1000 participants. Previous analyses were limited in terms of the number of participants or assessed the curriculum only from the lecturer’s perspective [2, 4, 5, 12, 13]. Our study focuses on the students’ perspective and illustrates the state of teaching RO in Germany on a federal level.

The discipline of RO is not as popular as other medical specialties [19, 20]. Therefore, the didactic task is twofold: to establish a basic understanding of major indications and treatments in RO for all students but also to foster motivated medical students to pursue a career in the field. Although only a minority of students are interested in pursuing a career in RO, a basic understanding in all medical students is seen as a major prerequisite for clinical practice and interdisciplinary oncology. In this regard, students agree on a broad curriculum covering basic science (radiation physics and biology) but also clinical indications and the physician–patient interaction.

There is an ongoing trend in transferring traditional knowledge-based medical curricula to competence-orientated practical education, which has been formalized on a national level within the NKLM [11, 21]. This is reflected by the free-text commentaries of the current survey. On the contrary, the expected level of competence at the end of medical studies, according to students, is limited to the analytical level, thus leaving room for improvement.

The amount of teaching is seen to be sufficient by most of the participants, and RO is not considered to be underrepresented. At the European level, an assessment from 19 countries revealed a median duration of RO teaching of 10 h, with a considerable range of 2–60 h [4]. Interestingly, this matches with the presented findings, as 1–5 h was considered an optimal duration for the four subdomains by many students; however, it contrasts with recommendations from the academic RO consortium of the DEGRO, which postulates 20 h of mandatory teaching [11]. This persistent underrepresentation may also be related to structural barriers such as limited faculty resources, scheduling constraints, and competing curricular priorities.

The qualitative analysis demonstrates a desire to increase practical teaching and patient-centered education but also to improve professional conversation skills. This stands in line with data from the literature, showing that empathic, patient-centered communication is pivotal and should be addressed in decisive RO teaching formats during medical school and residency [5, 13, 22–24].

Accordingly, RO is seen as a prerequisite for the role of medical expert (81.0%) and member of a team (67.4%) according to the roles predefined by the Canadian Medical Education Directives for Specialists (CanMED roles) [25, 26].

On a structural level, the extent of RO teaching as well as the semesters in which RO teaching takes place differ significantly, both at the national and at the international level [2, 4, 11]. Early integration of RO into the curriculum may help to generate a positive attitude towards the subject and create a longitudinal curriculum [12, 27]. An assessment from seven US-American universities demonstrates an improvement of RO knowledge longitudinally throughout the years of training, but knowledge gaps regarding its curative potential or the clinical duties of a radiation oncologist still persist [28]. In contrast, a recently published student assessment contradicts this concept by advocating for a focused curriculum within one semester only [13].

Hybrid concepts, as used in 13–27% of cases, may offer increased flexibility and on-demand content. Their implementation is desired by the students on a broader level reaching up to 55%. This is in line with previous data showing positive anticipation towards hybrid concepts [5, 13, 29, 30].

Innovative teaching concepts are valuable drivers to increase the attractiveness of RO beyond the curricular education and to attract future residents to the field. Previous publications highlight the successful implementation of student tumor boards, brachytherapy workshops, and interdisciplinary courses connecting anatomy, physiology, and biochemistry to radiology, nuclear medicine, and RO [7, 27, 31]. In the present analysis, a simulated interdisciplinary tumor board or a clerkship on the RO ward were the favorite elective teaching formats. Clerkships in RO have been shown to improve knowledge, counteract misconceptions, and increase interest in the specialty [12, 13, 32]. Implementation of field trips may also stimulate interest in the discipline [24]. Concrete implementation models include integration of RO into longitudinal oncology modules, student tumor boards as scalable teaching formats, hybrid electives to increase flexibility, and linking technical aspects (physics, biology) with clinical case discussions. These formats could be embedded into longitudinal oncology modules to ensure continuity across the curriculum. Hybrid electives may increase flexibility and scalability, while linking technical aspects such as physics and radiobiology directly to clinical case discussions can improve contextual learning.

With 65% of participants being female, this survey also reflects the growing percentage of female medical students and later physicians [33, 34]. This trend is currently not translated into the RO workforce, as shown by a US-American cross-sectional analysis with a constantly low female representation of 23.4 (2006) to 27.5% (2020) [35]—neglecting both the current parity of medical students and the important female contributions to RO in the past and present [36].

### Limitations

Despite the large number of participants, the study is limited by selection bias and representativeness. The observed enthusiasm likely reflects the views of a motivated subgroup with above-average interest in RO and should therefore be interpreted with caution when generalizing to the broader student population. Considering that typical German medical faculties have annual cohorts of ~250–400 students, the relative response rates per site were low. Faculties with strong RO teaching structures may also have been overrepresented, while institutions with minimal exposure may have contributed fewer responses. A more detailed subgroup analysis by semester, gender, or institution type was beyond the scope of this work and would carry a high risk of bias due to uneven participation across faculties. Future studies with balanced sampling may provide additional granularity.

## Conclusion

This nationwide survey highlights significant heterogeneity in undergraduate RO education across German medical faculties. While students acknowledge the relevance of RO in clinical practice, they report limited exposure and insufficient integration into curricula. The ongoing educational reforms may offer a timely opportunity to standardize and enhance RO teaching through competency-based, interdisciplinary, and practice-oriented formats. Systematic implementation will be essential to ensure that all graduates acquire core competencies in RO.

## Supplementary Information

The Supplementary Information provides a comprehensive description of the study design, survey development, recruitment process, and data analysis methods. In addition, it includes the complete survey instrument and detailed descriptive results for all questionnaire items, including demographic characteristics, teaching formats, content coverage, competency levels, and students’ perceptions of undergraduate radiation oncology education across German medical faculties. These data complement the main manuscript by offering full transparency and additional context for the reported findings.

## Abbreviations

ÄApprO: German Medical Licensing Regulations (*Ärztliche Approbationsordnung*)
CanMEDS: Canadian Medical Education Directives for Specialists
COVID-19: Coronavirus disease 2019
DEGRO: German Society for Radiation Oncology (*Deutsche Gesellschaft für Radioonkologie*)
ESTRO: European Society for Radiotherapy and Oncology
GDPR: General Data Protection Regulation
IRB: Institutional review board
NKLM: National Competence-Based Learning Objectives Catalogue for Medicine (*Nationaler Kompetenzbasierter Lernzielkatalog Medizin*)
RO: Radiation oncology
UE: Teaching units (*Unterrichtseinheiten*)
