# Supplementary material for: Shaping the future of radiation oncology education: results of a nationwide student survey from Germany
Source: Strahlenther Onkol. 2026 Jan 7;202(7):713–21. doi: 10.1007/s00066-025-02493-x (PMC13291027; doi:10.1007/s00066-025-02493-x)
Supplement: Supplementary file 1 — The Supplementary Information provides a comprehensive description of the study design, survey development, recruitment process, and data analysis methods. In addition, it includes the complete survey instrument and detailed descriptive results for all questionnaire items, including demographic characteristics, teaching formats, content coverage, competency levels, and students’ perceptions of undergraduate radiation oncology education across German medical faculties. These data complement the main manuscript by offering full transparency and additional context for the reported findings. [file 66_2025_2493_MOESM1_ESM.docx]

**Supplementary Material**

**Study Design and Methods**

This cross-sectional, web-based study aimed to evaluate the structure, content, and perceived quality of undergraduate radiation oncology (RO) education across all 38 German medical faculties. The survey was conducted over three academic terms, from the winter semester 2023/2024 to the winter semester 2024/2025. Eligible participants were medical students who had already completed the majority of their curriculum-based RO teaching.

The questionnaire was developed by members of the Working Group on Teaching (“Arbeitsgemeinschaft Lehre”) of the German Society for Radiation Oncology (DEGRO). This interdisciplinary team consisted of 12 RO educators from various medical faculties in Germany, most with several years of experience in undergraduate education. Early-career colleagues also contributed, providing perspectives more closely aligned with current student experiences.

The item development process followed a multi-stage, consensus-driven approach. Content was based on the *Nationaler Kompetenzbasierter Lernzielkatalog Medizin* (NKLM 2.0) and the draft framework of the revised German Medical Licensing Regulations (*Ärztliche Approbationsordnung*, ÄApprO). In addition, core educational priorities as defined by DEGRO—such as interdisciplinary oncology, clinical relevance, and fundamental knowledge in radiobiology and radiation physics – were incorporated. To ensure high question quality, the following criteria were applied: content and face validity, clarity and neutrality of wording, low cognitive load, and contextual appropriateness across different medical curricula.

A pilot version of the questionnaire was reviewed and tested by medical students and RO teaching faculty for comprehensibility, logic, and technical feasibility. The survey was then implemented using [UmfrageOnline](https://www.umfrageonline.com/) (Online®, Enuvo Inc., Switzerland) a General Data Protection Regulation (GDPR)-compliant platform enabling anonymized participation, flexible survey logic, and secure data handling.

**Survey Description**

The final survey consisted of 49 items covering seven major domains:

1. Demographics (e.g., university, semester, curriculum structure)
2. Evaluation of RO teaching (e.g., content, formats, perceived adequacy)
3. Preferred teaching formats and contexts (e.g., integration into interdisciplinary education)
4. Clinical and technical content (e.g., treatment planning, device handling)
5. Basic science topics (e.g., radiobiology, radiation physics, radiation protection)
6. Patient-related communication and bedside teaching
7. Student interest in RO and suggestions for improvement

The survey employed a mix of item formats: single- and multiple-choice, five-point Likert scales (from “strongly agree” to “strongly disagree”), dichotomous questions (yes/no), and open-ended text fields to capture qualitative feedback. Adaptive survey logic was used to tailor question pathways to prior responses – for example, adjusting based on students’ exposure to in-person vs. online RO instruction.

Question development followed established principles of high-quality educational survey design. Key quality standards included:

- **Content validity**, ensured through alignment with NKLM and the upcoming ÄApprO
- **Face validity**, through expert and student review during pilot testing
- **Clarity and neutrality**, by avoiding leading or ambiguous phrasing
- **Cognitive feasibility**, with a concise structure allowing completion within 10 minutes
- **Inclusivity**, through language accessible across diverse student backgrounds and curricula

**Recruitment and Consent**

Survey invitations were disseminated through multiple channels. These included outreach via student representative bodies (n=38; Fachschaften Humanmedizin), direct contact with RO teaching staff through university mailing lists, and announcements during national medical and oncology conferences. Participating departments were encouraged to announce the survey during RO lectures, with the majority incorporating it directly into their teaching activities. Monthly reminders and digital promotion via email and institutional platforms further supported recruitment.

Participation was voluntary and anonymous. At the beginning of the survey, participants were informed about the study’s purpose, data protection measures, and estimated completion time (approximately 9 minutes). Digital informed consent was obtained before access to the questionnaire was granted.

**Data Analysis**

Quantitative responses were analyzed using descriptive statistics (absolute and relative frequencies). Qualitative data from open-ended questions were subjected to thematic analysis by multiple reviewers (at least 3) to identify recurring themes and generate insights into students' perspectives on RO teaching.

**Ethical Considerations**

This study was conducted in accordance with the Declaration of Helsinki and complied with national data protection regulations. Ethical approval was obtained from the institutional review board (IRB) of University of Jena (approval number: 123456789).

**Importance Radiation Oncology for Understanding the Treatment of Oncology Patients**

87.65% fully or mostly agreed that radiation oncology is an important medical specialty. Most participants also considered radiotherapy a fundamental component of the medical curriculum (Figure 1)

**Figure 1**


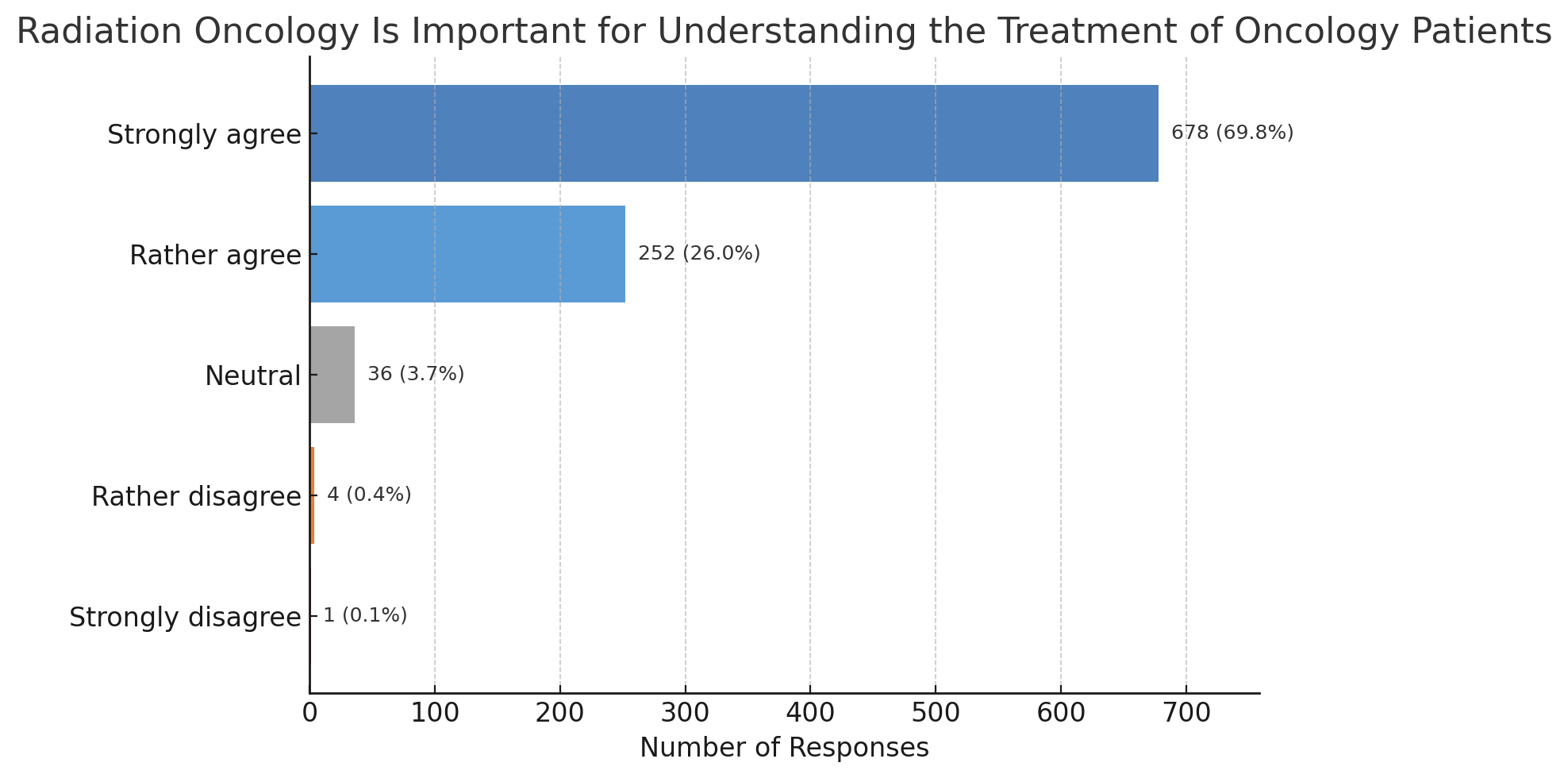


**Survey Report: Radiation Oncology Education**

| Where are you studying? (n = 1075) | Answer | Count | % | Value |
| --- | --- | --- | --- | --- |
|  | Aachen | 25 | 2.33 | 1 |
|  | augsburg | 1 | 0.09 | 2 |
|  | Berlin | 15 | 1.4 | 3 |
|  | Bielefeld | 0 | 0 | 4 |
|  | Bochum | 12 | 1.12 | 5 |
|  | Bonn-Siegen | 0 | 0 | 6 |
|  | Brandenburg (Brandenburg Theodor Fontane Medical School) | 10 | 0.93 | 7 |
|  | Dusseldorf | 0 | 0 | 8 |
|  | Duisburg-Essen | 24 | 2.23 | 9 |
|  | Dresden | 13 | 1.21 | 10 |
|  | Erlangen-Nuremberg | 3 | 0.28 | 11 |
|  | Eat | 5 | 0.47 | 12 |
|  | Frankfurt | 1 | 0.09 | 13 |
|  | Freiburg | 24 | 2.23 | 14 |
|  | Pour | 3 | 0.28 | 15 |
|  | Göttingen | 1 | 0.09 | 16 |
|  | Greifswald | 0 | 0 | 17 |
|  | Halle-Wittenberg | 5 | 0.47 | 18 |
|  | Hamburg | 1 | 0.09 | 19 |
|  | Hanover | 14 | 1.3 | 20 |
|  | Heidelberg | 1 | 0.09 | 21 |
|  | Homburg | 12 | 1.12 | 22 |
|  | Jena | 315 | 29.3 | 23 |
|  | Kassel (School of Medicine) | 0 | 0 | 24 |
|  | Kiel | 9 | 0.84 | 25 |
|  | Cologne | 133 | 12.37 | 26 |
|  | Leipzig | 2 | 0.19 | 27 |
|  | Lübeck | 16 | 1.49 | 28 |
|  | Magdeburg | 1 | 0.09 | 29 |
|  | Mainz | 262 | 24.37 | 30 |
|  | Mannheim | 8 | 0.74 | 31 |
|  | Marburg | 50 | 4.65 | 32 |
|  | Munich (LMU) | 4 | 0.37 | 33 |
|  | Munich (TUM) | 3 | 0.28 | 34 |
|  | Muenster | 66 | 6.14 | 35 |
|  | regensburg | 0 | 0 | 36 |
|  | Rostock | 10 | 0.93 | 37 |
|  | Tübingen | 5 | 0.47 | 38 |
|  | Ulm | 12 | 1.12 | 39 |
|  | Witten/Herdecke | 0 | 0 | 40 |
|  | Würzburg | 9 | 0.84 | 41 |
|  |  |  |  |  |
|  | Average |  |  | 24.96 |
|  | Standard Deviation |  |  | 8.06 |
|  |  |  |  |  |
| In which (regular) study phase are you currently? (n = 1088) | Answer | Count | % |  |
|  | 5th or 6th clinical semester | 440 | 40.44 |  |
|  | Practical year | 63 | 5.79 |  |
|  | approved for a maximum of one year | 14 | 1.29 |  |
|  | Other: | 571 | 52.48 |  |
|  |  |  |  |  |
| What type of degree program/curriculum do you have at your location? (n = 1090) | Answer | Count | % |  |
|  | Standard course of study (pre-clinical/clinical) | 883 | 81.01 |  |
|  | Model study program | 200 | 18.35 |  |
|  | Other: | 7 | 0.64 |  |
|  |  |  |  |  |
| What is your gender? (n = 1094) | Answer | Count | % | Value |
|  | female | 716 | 65.45 | 1 |
|  | masculine | 370 | 33.82 | 2 |
|  | diverse | 4 | 0.37 | 3 |
|  | not specified | 4 | 0.37 | 4 |
|  |  |  |  |  |
|  | Average |  |  | 1.36 |
|  | Standard Deviation |  |  | 0.51 |
|  |  |  |  |  |
| Did your radiotherapy teaching primarily take place at a time when face-to-face teaching was significantly reduced due to the coronavirus pandemic? (n = 1087) | Answer | Count | % | Value |
|  | Yes | 312 | 28.7 | 1 |
|  | No | 775 | 71.3 | 2 |
|  |  |  |  |  |
|  | Average |  |  | 1.71 |
|  | Standard Deviation |  |  | 0.45 |
|  |  |  |  |  |
| Do I know the difference between radiotherapy and radiology and nuclear medicine? (n = 976) | Answer | Count | % | Value |
|  | I totally agree | 487 | 49.9 | 1 |
|  | tend to agree | 318 | 32.58 | 2 |
|  | partly/partly | 116 | 11.89 | 3 |
|  | rather disagree | 51 | 5.23 | 4 |
|  | strongly disagree | 4 | 0.41 | 5 |
|  |  |  |  |  |
|  | Average |  |  | 1.74 |
|  | Standard Deviation |  |  | 0.89 |
|  |  |  |  |  |
| Radiotherapy is most commonly used for the following diseases: (n = 973) | Answer | Count | % | Value |
|  | benign diseases (e.g. proliferative/inflammatory) diseases | 4 | 0.41 | 1 |
|  | malignant neoplasms | 750 | 77.08 | 2 |
|  | in approximately equal proportions for benign and malignant diseases | 202 | 20.76 | 3 |
|  | other | 1 | 0.1 | 4 |
|  | unclear | 16 | 1.64 | 5 |
|  |  |  |  |  |
|  | Average |  |  | 2.25 |
|  | Standard Deviation |  |  | 0.55 |
|  |  |  |  |  |
| I consider radiation therapy to be an important subject, the basics of which medical graduates should have a solid understanding. (n = 973) | Answer | Count | % | Value |
|  | I totally agree | 460 | 47.28 | 1 |
|  | tend to agree | 393 | 40.39 | 2 |
|  | partly/partly | 106 | 10.89 | 3 |
|  | rather disagree | 13 | 1.34 | 4 |
|  | strongly disagree | 1 | 0.1 | 5 |
|  |  |  |  |  |
|  | Average |  |  | 1.67 |
|  | Standard Deviation |  |  | 0.73 |
|  |  |  |  |  |
| Radiotherapy is important for the overall understanding of the treatment of cancer patients. (n = 972) | Answer | Count | % | Value |
|  | I totally agree | 679 | 69.86 | 1 |
|  | tend to agree | 252 | 25.93 | 2 |
|  | partly/partly | 36 | 3.7 | 3 |
|  | rather disagree | 4 | 0.41 | 4 |
|  | strongly disagree | 1 | 0.1 | 5 |
|  |  |  |  |  |
|  | Average |  |  | 1.35 |
|  | Standard Deviation |  |  | 0.58 |
|  |  |  |  |  |
| Radiation therapy was adequately taught at our faculty overall. (n = 971) | Answer | Count | % | Value |
|  | I totally agree | 233 | 24 | 1 |
|  | tend to agree | 320 | 32.96 | 2 |
|  | partly/partly | 247 | 25.44 | 3 |
|  | rather disagree | 141 | 14.52 | 4 |
|  | strongly disagree | 30 | 3.09 | 5 |
|  |  |  |  |  |
|  | Average |  |  | 2.4 |
|  | Standard Deviation |  |  | 1.09 |
|  |  |  |  |  |
| Radiotherapy was underrepresented in our teaching compared to other oncology disciplines. (n = 966) | Answer | Count | % | Value |
|  | I totally agree | 109 | 11.28 | 1 |
|  | tend to agree | 260 | 26.92 | 2 |
|  | partly/partly | 272 | 28.16 | 3 |
|  | rather disagree | 267 | 27.64 | 4 |
|  | strongly disagree | 58 | 6 | 5 |
|  |  |  |  |  |
|  | Average |  |  | 2.9 |
|  | Standard Deviation |  |  | 1.11 |
|  |  |  |  |  |
| In the future, radiotherapy should most likely be taught in the following context: (n = 974) | Answer | Count | % |  |
|  | Imaging procedures (together with radiology/nuclear medicine) | 330 | 33.88 |  |
|  | independent subject | 157 | 16.12 |  |
|  | interdisciplinary oncology | 451 | 46.3 |  |
|  | only accompanying other subjects | 30 | 3.08 |  |
|  | other | 6 | 0.62 |  |
|  |  |  |  |  |
| Radiotherapy indications and specific therapies are/were taught in our department primarily in the following teaching format (multiple choices possible): (n = 878) | Answer | Count | % |  |
|  | Lectures | 634 | 72.21 |  |
|  | Seminars and internships | 658 | 74.94 |  |
|  | exclusively e-learning based (e.g. as online video, online course) | 51 | 5.81 |  |
|  | Hybrid format (e.g. combination of e-learning-based pre-/post-processing and seminar/internship) | 237 | 26.99 |  |
|  | not at all | 3 | 0.34 |  |
|  | I can't remember | 38 | 4.33 |  |
|  | other: | 14 | 1.59 |  |
|  |  |  |  |  |
| Radiotherapy indications and specific therapies should most likely be taught in the following teaching format: (n = 875) | Answer | Count | % |  |
|  | lecture | 174 | 19.89 |  |
|  | Seminar or internship | 673 | 76.91 |  |
|  | not at all | 4 | 0.46 |  |
|  | other: | 24 | 2.74 |  |
|  |  |  |  |  |
| Radiotherapy indications and specific therapies should be communicated in the following form: (n = 875) | Answer | Count | % |  |
|  | In-person event | 362 | 41.37 |  |
|  | hybrid format (both as a face-to-face event and e-learning-based (e.g. as an online video, online course)) | 479 | 54.74 |  |
|  | exclusively e-learning based (e.g. as online video, online course) | 25 | 2.86 |  |
|  | not at all | 3 | 0.34 |  |
|  | other: | 6 | 0.69 |  |
|  |  |  |  |  |
| Radiotherapy indications and specific therapies are/were adequately taught at our faculty. (n = 872) | Answer | Count | % | Value |
|  | I totally agree | 153 | 17.55 | 1 |
|  | tend to agree | 378 | 43.35 | 2 |
|  | partly/partly | 222 | 25.46 | 3 |
|  | rather disagree | 105 | 12.04 | 4 |
|  | strongly disagree | 14 | 1.61 | 5 |
|  |  |  |  |  |
|  | Average |  |  | 2.37 |
|  | Standard Deviation |  |  | 0.96 |
|  |  |  |  |  |
| Radiotherapy indications and specific therapies should be taught together in approximately the following amount of time (in teaching units, 1 teaching unit = 45 min): (n = 856) | Answer | Count | % | Value |
|  | 0 UE | 2 | 0.23 | 1 |
|  | 1 - 5 teaching units | 328 | 38.32 | 2 |
|  | 6 - 10 teaching units | 363 | 42.41 | 3 |
|  | 11 - 15 teaching units | 116 | 13.55 | 4 |
|  | 15 - 20 teaching units | 36 | 4.21 | 5 |
|  | > 20 units | 11 | 1.29 | 6 |
|  |  |  |  |  |
|  | Average |  |  | 2.87 |
|  | Standard Deviation |  |  | 0.89 |
|  |  |  |  |  |
| Radiotherapy indications and specific therapies should be mastered by students at the end of their studies to the following level of competence: (n = 866) | Answer | Count | % | Value |
|  | Factual knowledge (naming and describing facts) | 121 | 13.97 | 1 |
|  | Action and reasoning knowledge (explaining facts and relationships) | 668 | 77.14 | 2 |
|  | Action competence (carry out under supervision) | 43 | 4.97 | 3 |
|  | Advanced action competence (carry out actions independently and appropriately in accordance with the situation, knowing the consequences) | 30 | 3.46 | 4 |
|  | none at all | 4 | 0.46 | 5 |
|  |  |  |  |  |
|  | Average |  |  | 1.99 |
|  | Standard Deviation |  |  | 0.61 |
|  |  |  |  |  |
| Radiotherapy indications and specific therapies should be taught in the following subject context: (n = 868) | Answer | Count | % |  |
|  | in the context of other radiotherapy topics in the subject of radiotherapy | 172 | 19.82 |  |
|  | in the context of other oncological disciplines (oncological interdisciplinary) | 565 | 65.09 |  |
|  | in the context of imaging disciplines (radiology, nuclear medicine) | 126 | 14.52 |  |
|  | not at all | 2 | 0.23 |  |
|  | other: | 3 | 0.35 |  |
|  |  |  |  |  |
| Patient-related teaching and communication skills in radiotherapy are/were taught in our department primarily in the following teaching format (multiple choices possible): (n = 785) | Answer | Count | % |  |
|  | Examination course (bedside teaching or similar) | 106 | 13.5 |  |
|  | Seminar or internship | 392 | 49.94 |  |
|  | Hybrid format (e.g. combination of e-learning based pre-/post-processing and examination course) | 124 | 15.8 |  |
|  | exclusively e-learning based (e.g. as online video, online course) | 34 | 4.33 |  |
|  | not at all | 239 | 30.45 |  |
|  | I can't remember | 81 | 10.32 |  |
|  | other: | 16 | 2.04 |  |
|  |  |  |  |  |
| Patient-focused teaching and communication skills in radiotherapy should most likely be taught in the following teaching format: (n = 781) | Answer | Count | % |  |
|  | Examination course (bedside teaching or similar) | 379 | 48.53 |  |
|  | Seminar or internship | 346 | 44.3 |  |
|  | not at all | 45 | 5.76 |  |
|  | other: | 11 | 1.41 |  |
|  |  |  |  |  |
| Patient-related teaching and communication skills in radiotherapy should most likely be taught in the following form: (n = 779) | Answer | Count | % |  |
|  | In-person event | 497 | 63.8 |  |
|  | hybrid format (both as a face-to-face event and e-learning-based (e.g. as an online video, online course)) | 221 | 28.37 |  |
|  | exclusively e-learning based (e.g. as online video, online course) | 21 | 2.7 |  |
|  | not at all | 36 | 4.62 |  |
|  | other: | 4 | 0.51 |  |
|  |  |  |  |  |
| Patient-focused teaching and communication skills in radiation therapy are/were adequately taught at our faculty. (n = 774) | Answer | Count | % | Value |
|  | I totally agree | 57 | 7.36 | 1 |
|  | tend to agree | 148 | 19.12 | 2 |
|  | partly/partly | 186 | 24.03 | 3 |
|  | rather disagree | 272 | 35.14 | 4 |
|  | strongly disagree | 111 | 14.34 | 5 |
|  |  |  |  |  |
|  | Average |  |  | 3.3 |
|  | Standard Deviation |  |  | 1.15 |
|  |  |  |  |  |
| Patient-related teaching and communication skills in radiotherapy should be taught together in approximately the following time frame (in teaching units, 1 teaching unit = 45 min): (n = 759) | Answer | Count | % | Value |
|  | 0 UE | 53 | 6.98 | 1 |
|  | 1 - 5 teaching units | 541 | 71.28 | 2 |
|  | 6 - 10 teaching units | 134 | 17.65 | 3 |
|  | 11 - 15 teaching units | 19 | 2.5 | 4 |
|  | 15 - 20 teaching units | 8 | 1.05 | 5 |
|  | > 20 units | 4 | 0.53 | 6 |
|  |  |  |  |  |
|  | Average |  |  | 2.21 |
|  | Standard Deviation |  |  | 0.69 |
|  |  |  |  |  |
| Patient-related teaching and communication skills in radiotherapy should be mastered by students at the end of their studies to the following level of competence: (n = 774) | Answer | Count | % | Value |
|  | Factual knowledge (naming and describing facts) | 96 | 12.4 | 1 |
|  | Action and reasoning knowledge (explaining facts and relationships) | 372 | 48.06 | 2 |
|  | Action competence (carry out under supervision) | 240 | 31.01 | 3 |
|  | Advanced action competence (carry out actions independently and appropriately in accordance with the situation, knowing the consequences) | 48 | 6.2 | 4 |
|  | none at all | 18 | 2.33 | 5 |
|  |  |  |  |  |
|  | Average |  |  | 2.38 |
|  | Standard Deviation |  |  | 0.86 |
|  |  |  |  |  |
| Patient-centered teaching and communication skills in radiotherapy should be taught in the following subject context: (n = 774) | Answer | Count | % |  |
|  | in the context of other radiotherapy topics in the subject of radiotherapy | 179 | 23.13 |  |
|  | in the context of other oncological disciplines (oncological interdisciplinary) | 494 | 63.82 |  |
|  | in the context of imaging disciplines (radiology, nuclear medicine) | 81 | 10.47 |  |
|  | not at all | 20 | 2.58 |  |
|  | other: | 0 | 0 |  |
|  |  |  |  |  |
| Scientific fundamentals (radiation biology, radiation physics, radiation protection) are/were taught in the following teaching format (multiple choices possible): (n = 759) | Answer | Count | % |  |
|  | lecture | 554 | 72.99 |  |
|  | Seminar or internship | 366 | 48.22 |  |
|  | Exclusively e-learning-based (e.g. as an online video, online course or online seminar) | 65 | 8.56 |  |
|  | Hybrid format (e.g. combination of individual e-learning-based pre-/post-processing and seminar/internship) | 142 | 18.71 |  |
|  | several of the listed here | 32 | 4.22 |  |
|  | not at all | 17 | 2.24 |  |
|  | I can't remember | 41 | 5.4 |  |
|  | other: | 6 | 0.79 |  |
|  |  |  |  |  |
| Scientific fundamentals (radiation biology, radiation physics, radiation protection) should most likely be taught in the following teaching format: (n = 756) | Answer | Count | % |  |
|  | lecture | 488 | 64.55 |  |
|  | Seminar or internship | 233 | 30.82 |  |
|  | not at all | 24 | 3.17 |  |
|  | other: | 11 | 1.46 |  |
|  |  |  |  |  |
| Scientific fundamentals (radiation biology, radiation physics, radiation protection) should be taught in the following form: (n = 756) | Answer | Count | % |  |
|  | In-person event | 173 | 22.88 |  |
|  | hybrid format (both as a face-to-face event and e-learning-based (e.g. as an online video, online course)) | 414 | 54.76 |  |
|  | exclusively e-learning based (e.g. as online video, online course) | 145 | 19.18 |  |
|  | not at all | 22 | 2.91 |  |
|  | other: | 2 | 0.26 |  |
|  |  |  |  |  |
| Fundamentals of natural science (radiation biology, radiation physics, radiation protection) are/were adequately taught at our faculty. (n = 748) | Answer | Count | % | Value |
|  | I totally agree | 196 | 26.2 | 1 |
|  | tend to agree | 302 | 40.37 | 2 |
|  | partly/partly | 172 | 22.99 | 3 |
|  | rather disagree | 67 | 8.96 | 4 |
|  | strongly disagree | 11 | 1.47 | 5 |
|  |  |  |  |  |
|  | Average |  |  | 2.19 |
|  | Standard Deviation |  |  | 0.97 |
|  |  |  |  |  |
| Scientific fundamentals (radiation biology, radiation physics, radiation protection) should be taught together in approximately the following time frame (in teaching units, 1 teaching unit = 45 min): (n = 731) | Answer | Count | % | Value |
|  | 0 UE | 27 | 3.69 | 1 |
|  | 1 - 5 teaching units | 582 | 79.62 | 2 |
|  | 6 - 10 teaching units | 92 | 12.59 | 3 |
|  | 11 - 15 teaching units | 20 | 2.74 | 4 |
|  | 15 - 20 teaching units | 6 | 0.82 | 5 |
|  | > 20 units | 4 | 0.55 | 6 |
|  |  |  |  |  |
|  | Average |  |  | 2.19 |
|  | Standard Deviation |  |  | 0.63 |
|  |  |  |  |  |
| Scientific fundamentals (radiation biology, radiation physics, radiation protection) should be mastered by students at the end of their studies to the following level of competence: (n = 749) | Answer | Count | % | Value |
|  | Factual knowledge (naming and describing facts) | 400 | 53.4 | 1 |
|  | Action and reasoning knowledge (explaining facts and relationships) | 289 | 38.58 | 2 |
|  | Action competence (carry out under supervision) | 29 | 3.87 | 3 |
|  | Advanced action competence (carry out actions independently and appropriately in accordance with the situation, knowing the consequences) | 18 | 2.4 | 4 |
|  | none at all | 13 | 1.74 | 5 |
|  |  |  |  |  |
|  | Average |  |  | 1.6 |
|  | Standard Deviation |  |  | 0.82 |
|  |  |  |  |  |
| Scientific fundamentals (radiation biology, radiation physics, radiation protection) should be taught in the following subject context: (n = 746) | Answer | Count | % |  |
|  | in the context of other radiotherapy topics in the subject of radiotherapy | 266 | 35.66 |  |
|  | in the context of other oncological disciplines (oncological interdisciplinary) | 167 | 22.39 |  |
|  | in the context of imaging disciplines (radiology, nuclear medicine) | 288 | 38.61 |  |
|  | not at all | 19 | 2.55 |  |
|  | other: | 6 | 0.8 |  |
|  |  |  |  |  |
| Clinical and technical fundamentals of radiotherapy (treatment planning, treatment execution, equipment knowledge) are/were taught in the following teaching format (multiple choices possible): (n = 720) | Answer | Count | % |  |
|  | lecture | 405 | 56.25 |  |
|  | Seminar or internship | 445 | 61.81 |  |
|  | Exclusively e-learning-based (e.g. as an online video, online course or online seminar) | 34 | 4.72 |  |
|  | Hybrid format (e.g. combination of individual e-learning-based pre-/post-processing and seminar/internship) | 96 | 13.33 |  |
|  | several of the listed here | 17 | 2.36 |  |
|  | not at all | 45 | 6.25 |  |
|  | I can't remember | 42 | 5.83 |  |
|  | other: | 4 | 0.56 |  |
|  |  |  |  |  |
| Clinical and technical fundamentals of radiotherapy (treatment planning, treatment execution, equipment knowledge) should be taught in the following teaching format: (n = 713) | Answer | Count | % |  |
|  | lecture | 235 | 32.96 |  |
|  | Seminar or internship | 451 | 63.25 |  |
|  | not at all | 19 | 2.66 |  |
|  | other: | 8 | 1.12 |  |
|  |  |  |  |  |
| Clinical and technical fundamentals of radiotherapy (treatment planning, treatment execution, equipment knowledge) should be taught in the following form: (n = 716) | Answer | Count | % |  |
|  | In-person event | 266 | 37.15 |  |
|  | hybrid format (both as a face-to-face event and e-learning-based (e.g. as an online video, online course)) | 358 | 50 |  |
|  | exclusively e-learning based (e.g. as online video, online course) | 71 | 9.92 |  |
|  | not at all | 17 | 2.37 |  |
|  | other: | 4 | 0.56 |  |
|  |  |  |  |  |
| Clinical and technical fundamentals of radiotherapy (treatment planning, treatment execution, and equipment knowledge) are/were adequately taught at our faculty. (n = 711) | Answer | Count | % | Value |
|  | I totally agree | 167 | 23.49 | 1 |
|  | tend to agree | 276 | 38.82 | 2 |
|  | partly/partly | 152 | 21.38 | 3 |
|  | rather disagree | 90 | 12.66 | 4 |
|  | strongly disagree | 26 | 3.66 | 5 |
|  |  |  |  |  |
|  | Average |  |  | 2.34 |
|  | Standard Deviation |  |  | 1.08 |
|  |  |  |  |  |
| Clinical and technical fundamentals of radiotherapy (treatment planning, treatment execution, equipment knowledge) should be taught together in approximately the following time frame (in teaching units, 1 teaching unit = 45 min): (n = 687) | Answer | Count | % | Value |
|  | 0 UE | 16 | 2.33 | 1 |
|  | 1 - 5 teaching units | 568 | 82.68 | 2 |
|  | 6 - 10 teaching units | 86 | 12.52 | 3 |
|  | 11 - 15 teaching units | 12 | 1.75 | 4 |
|  | 15 - 20 teaching units | 4 | 0.58 | 5 |
|  | > 20 units | 1 | 0.15 | 6 |
|  |  |  |  |  |
|  | Average |  |  | 2.16 |
|  | Standard Deviation |  |  | 0.52 |
|  |  |  |  |  |
| Clinical and technical fundamentals of radiotherapy (treatment planning, treatment execution, equipment knowledge) should be mastered by students at the end of their studies to the following level of competence: (n = 717) | Answer | Count | % | Value |
|  | Factual knowledge (naming and describing facts) | 300 | 41.84 | 1 |
|  | Action and reasoning knowledge (explaining facts and relationships) | 343 | 47.84 | 2 |
|  | Action competence (carry out under supervision) | 50 | 6.97 | 3 |
|  | Advanced action competence (to act independently and appropriately in a situation-based manner, aware of the consequences) | 11 | 1.53 | 4 |
|  | none at all | 13 | 1.81 | 5 |
|  |  |  |  |  |
|  | Average |  |  | 1.74 |
|  | Standard Deviation |  |  | 0.8 |
|  |  |  |  |  |
| Clinical and technical principles of radiotherapy (treatment planning, treatment execution, equipment knowledge) should be taught in the following subject context: (n = 714) | Answer | Count | % |  |
|  | in the context of other radiotherapy topics in the subject of radiotherapy | 290 | 40.62 |  |
|  | in the context of other oncological disciplines (oncological interdisciplinary) | 213 | 29.83 |  |
|  | in the context of imaging disciplines (radiology, nuclear medicine) | 188 | 26.33 |  |
|  | not at all | 21 | 2.94 |  |
|  | other: | 2 | 0.28 |  |
|  |  |  |  |  |
| I find radiation therapy fundamentally interesting. (n = 720) | Answer | Count | % | Value |
|  | I totally agree | 188 | 26.11 | 1 |
|  | tend to agree | 280 | 38.89 | 2 |
|  | partly/partly | 164 | 22.78 | 3 |
|  | rather disagree | 77 | 10.69 | 4 |
|  | strongly disagree | 11 | 1.53 | 5 |
|  |  |  |  |  |
|  | Average |  |  | 2.23 |
|  | Standard Deviation |  |  | 1 |
|  |  |  |  |  |
| I am/would be interested in these optional radiotherapy courses (multiple choices possible): (n = 718) | Answer | Count | % |  |
|  | Internship/PJ/Observation | 199 | 27.72 |  |
|  | Elective subject/block internship | 326 | 45.4 |  |
|  | Doctoral thesis/scientific work | 130 | 18.11 |  |
|  | Interdisciplinary oncology involving radiotherapy (e.g. tumor board simulation) | 435 | 60.58 |  |
|  | none | 129 | 17.97 |  |
|  | other: | 6 | 0.84 |  |
|  |  |  |  |  |
| How strong is/would you have been in general your interest in elective teaching opportunities in radiotherapy (e.g., clinical internship, internship, etc.)? (n = 719) | Answer | Count | % | Value |
|  | very large | 57 | 7.93 | 1 |
|  | large | 163 | 22.67 | 2 |
|  | mediocre | 305 | 42.42 | 3 |
|  | small amount | 141 | 19.61 | 4 |
|  | very low | 53 | 7.37 | 5 |
|  |  |  |  |  |
|  | Average |  |  | 2.96 |
|  | Standard Deviation |  |  | 1.02 |
|  |  |  |  |  |
| I find/found the elective courses (e.g., clinical internship, practical year, internships, elective courses) in the field of radiation therapy at our faculty sufficient. (n = 704) | Answer | Count | % | Value |
|  | I totally agree | 157 | 22.3 | 1 |
|  | tend to agree | 285 | 40.48 | 2 |
|  | partly/partly | 192 | 27.27 | 3 |
|  | rather disagree | 58 | 8.24 | 4 |
|  | strongly disagree | 12 | 1.7 | 5 |
|  |  |  |  |  |
|  | Average |  |  | 2.27 |
|  | Standard Deviation |  |  | 0.95 |
|  |  |  |  |  |
| The current draft of the new licensing regulations provides for a core curriculum (70%) and an individually selectable specialization (30%) at the faculties. How interested are/would you be in choosing a specialization that also includes more intensive coverage of radiotherapy? (n = 717) | Answer | Count | % | Value |
|  | very large | 46 | 6.42 | 1 |
|  | large | 176 | 24.55 | 2 |
|  | mediocre | 309 | 43.1 | 3 |
|  | small amount | 130 | 18.13 | 4 |
|  | very low | 56 | 7.81 | 5 |
|  |  |  |  |  |
|  | Average |  |  | 2.96 |
|  | Standard Deviation |  |  | 1 |
|  |  |  |  |  |
| Radiotherapy is particularly important for physicians in their role as… (multiple choices possible) (n = 710) | Answer | Count | % |  |
|  | Medical expert | 576 | 81.13 |  |
|  | scholar | 138 | 19.44 |  |
|  | Communicator | 269 | 37.89 |  |
|  | member of a team | 479 | 67.46 |  |
|  | Health Advisor & Advocate | 278 | 39.15 |  |
|  | Responsible person & manager | 232 | 32.68 |  |
|  | Professionally acting person | 355 | 50 |  |
|  |  |  |  |  |
| Radiation therapy is a field I could fundamentally imagine working in. (n = 715) | Answer | Count | % | Value |
|  | I totally agree | 60 | 8.39 | 1 |
|  | tend to agree | 107 | 14.97 | 2 |
|  | partly/partly | 175 | 24.48 | 3 |
|  | rather disagree | 246 | 34.41 | 4 |
|  | strongly disagree | 127 | 17.76 | 5 |
|  |  |  |  |  |
|  | Average |  |  | 3.38 |
|  | Standard Deviation |  |  | 1.18 |
|  |  |  |  |  |
| What could help increase my interest in radiation therapy? (n = 667) | Answer | Count | % |  |
|  | I can't think of anything | 487 | 73.01 |  |
|  | Free text input | 180 | 26.99 |  |
|  |  |  |  |  |
| I currently assess my knowledge and skills in radiotherapy as follows: (n = 715) | Answer | Count | % | Value |
|  | very large | 9 | 1.26 | 1 |
|  | large | 83 | 11.61 | 2 |
|  | mediocre | 435 | 60.84 | 3 |
|  | small amount | 145 | 20.28 | 4 |
|  | very low | 43 | 6.01 | 5 |
|  |  |  |  |  |
|  | Average |  |  | 3.18 |
|  | Standard Deviation |  |  | 0.76 |

*Table automatically translated from German via Google Translator*
